# Effectiveness of Flippits and Virtual Reality Therapy on Pain and Anxiety Among Children Undergoing Painful Procedures

**DOI:** 10.7759/cureus.17134

**Published:** 2021-08-12

**Authors:** Sekkulandai K Mohanasundari, Valalahalli A Raghu, Joyce Joseph, Remiya Mohan, Suresh Sharma

**Affiliations:** 1 College of Nursing, All India Institute of Medical Sciences, Jodhpur, IND

**Keywords:** virtual reality, flippit, distraction card, pain, anxiety

## Abstract

Introduction

Pain experienced by children during painful procedures may cause stress, fear, and anxiety. Currently, a number of interventions are used to reduce pain perception during medical procedures and distraction therapy is one of the most commonly used interventions.

Method

A randomized control trial was conducted among 105 children aged between three years and 12 years undergoing painful procedures such as intravenous cannulation, blood sampling, and injections to evaluate the effect of flippits and virtual reality therapy (VRT) on pain and anxiety*.* Through a computerized random approach, 35 samples were allotted to each group. Experimental group -1 received VRT, experimental group -2 received flippit (distraction card) therapy during painful procedures, and the control group received the conventional intervention. Standard tools were used to assess the pain and anxiety.

Result

Total 128 children were admitted to the ward and 23 were not included in the study for various reasons. Total 105 children undergone randomization to three groups, 35 in each group. All were analyzed for primary and secondary outcomes. After adjusting for confounding factors using multiple logistic regression, it was found that pain scores of VRT and flippit groups were less than the control group (aOR, 95% CI 0.635, 0.504-0.799, P = 0.000 and aOR, 95% CI 0.705, 0.572-0.868, P = 0.001, respectively) and no difference was observed between VRT and Flippit group (aOR, 95% CI; 0.901, 0.723 - 1.123, P 0.353). Flippit group perceived less intensity of pain compared to control group (aOR, 95% CI 0.542, 0.322-0.912, P = 0.021) and children received VRT perceived less intensity of pain than both control and flippit groups of children (aOR, 95% CI 0.258, 0.132-0.503, P = 0.000 and aOR, 95% CI 0.476, 0.252-0.900, respectively). Children received VRT and flippit therapy perceived less anxiety compared to control group (aOR, 95% CI 0.589, 0.348-0.999, P = 0.050 and aOR, 95% CI 0.385, 0.217-0.682, P = 0.001, respectively). But, there was no difference between VRT and flippit groups (aOR, 95% CI 1.532, 0.940-2.498, P = 0.087).

Conclusion

Flippit therapy and virtual reality therapy were better than conventional therapy in reducing the perception of anxiety and pain in children, aged three to 12 years, undergone painful procedures. Virtual reality therapy had an edge over flippit therapy in reducing the worst hurt.

## Introduction

Pain is a physical and emotional experience perceived and processed by the brain. It is sometimes unavoidable by the part of medical interventions to inflict pain during blood sampling, vaccination, medication, etc. More than 87% of children experience pain during hospital admission [1]. Pain is one of the under-recognized and under-treated problems in children. As per New Joint Commission on Accreditation of Healthcare Organizations (JCAHO) regulations, pain is to be considered as the “fifth vital sign.” Children experiencing pain have an increased risk of anxiety, distress, and psychological disturbances.

Non-opioid analgesics (NSAIDs) and acetaminophen are the common pharmacological measures for pain management of children. Opioid analgesics are rarely used. Adverse effects due to NSAIDs are inhibition of bone growth and healing, gastritis, acute kidney injury, platelet dysfunction, and exacerbation of acute asthmatic attack [2]. Opioid use is restricted to prolonged moderate to severe pain or chronic pain. Sedation, respiratory depression, euphoria, physical dependency, and endocrine disturbances are the major adverse effects due to opioid use [2]. Works of literature support the effectiveness of both pharmacological and non-pharmacological techniques for pain management during invasive procedures [3]. In addition, now getting evidence for cognitive interventions in the management of pain during an invasive procedure. Systematic review and meta-analysis done by Birnie et al. support the efficacy of distraction therapy and hypnotic therapy in reducing pain and distress during painful procedures [4].

The distraction techniques act on the cognitive level to divert the children’s attention from the painful stimuli. Distraction could be done in an active way which includes play or passive way includes visual or auditory stimuli using toys, music, movies, virtual reality therapy, video games, television, blowing bubbles, and flippit cards which help to distract the child's attention from the painful stimuli. Flippit therapy can significantly reduce the pain associated with painful procedures such as blood sampling in children [5].

Virtual reality is a computer technology that creates an artificial three-dimensional simulated environment that might offer distraction, as it immerses the patient in another world and involves multiple senses [6]. Virtual reality consists of a head-mounted display and a thick pair of goggles that are connected to either a computer or a cell phone. Patients can actively or passively participate in numerous potential programs. Virtual reality is thought to reduce pain by directing children’s attention into the virtual world, leaving less attention available to process incoming neural signals from pain receptors [7].

In a study done by Hoffman et al., video game was incorporated in virtual reality therapy in the pain management for burns patients and found that 35% of patients perceived pain who received virtual reality therapy with standard pharmacological treatment and 50% of patients perceived pain who received pharmacologic treatment alone [8]. The beneficial effect of virtual reality therapy was proved in reducing anxiety, posttraumatic stress disorder, and pain during painful procedures [5].

It is not uncommon for parents to postpone vaccination injection for their children owing to concerns about discomfort [9]. Studies proved the benefits of virtual reality therapy in the reduction of acute and chronic pain among children [6]. Also recently, distraction cards (flippits) were found to be effective in reducing procedural pain and anxiety in children during blood sampling.

At present with the best of our knowledge, only one randomized controlled trial (done by Erdogan et al.) is available on comparing flippit therapy and virtual reality therapy in controlling pain and anxiety. This study was done in children aged seven to 12 years, undergone venipuncture and found that both flippit therapy and virtual reality therapy were effective in controlling pain and anxiety compared to the control group, but there was no significant difference between them [3]. We aimed to explore the extended use of flippit therapy and virtual reality therapy in children aged three to 12 years and who undergo intravenous cannulation, blood sampling and injections. 

## Materials and methods

Study design, setting, and duration

This study was an open-label, three-arm, parallel design, randomized control trial conducted in a tertiary care hospital, All Institute of Medical Sciences, Jodhpur. This study was approved by Institute Ethical Committee (AIIMS/IEC/2021/3450) and registered in the Clinical Trial Registry of India (CTRI number - CTRI/2021/03/032300) before commencement. Enrollment was done from March 2021 to May 2021. Informed assent was obtained from children aged >7 years and consent from accompanying person of children aged ≤7 years.

Methods

We aimed to evaluate the effects of flippit therapy and virtual reality therapy (VRT) on the pain and anxiety of the children during painful procedures compared to conventional measures (control group). 

Inclusion and Exclusion Criteria of Study Population

**Figure 1:**
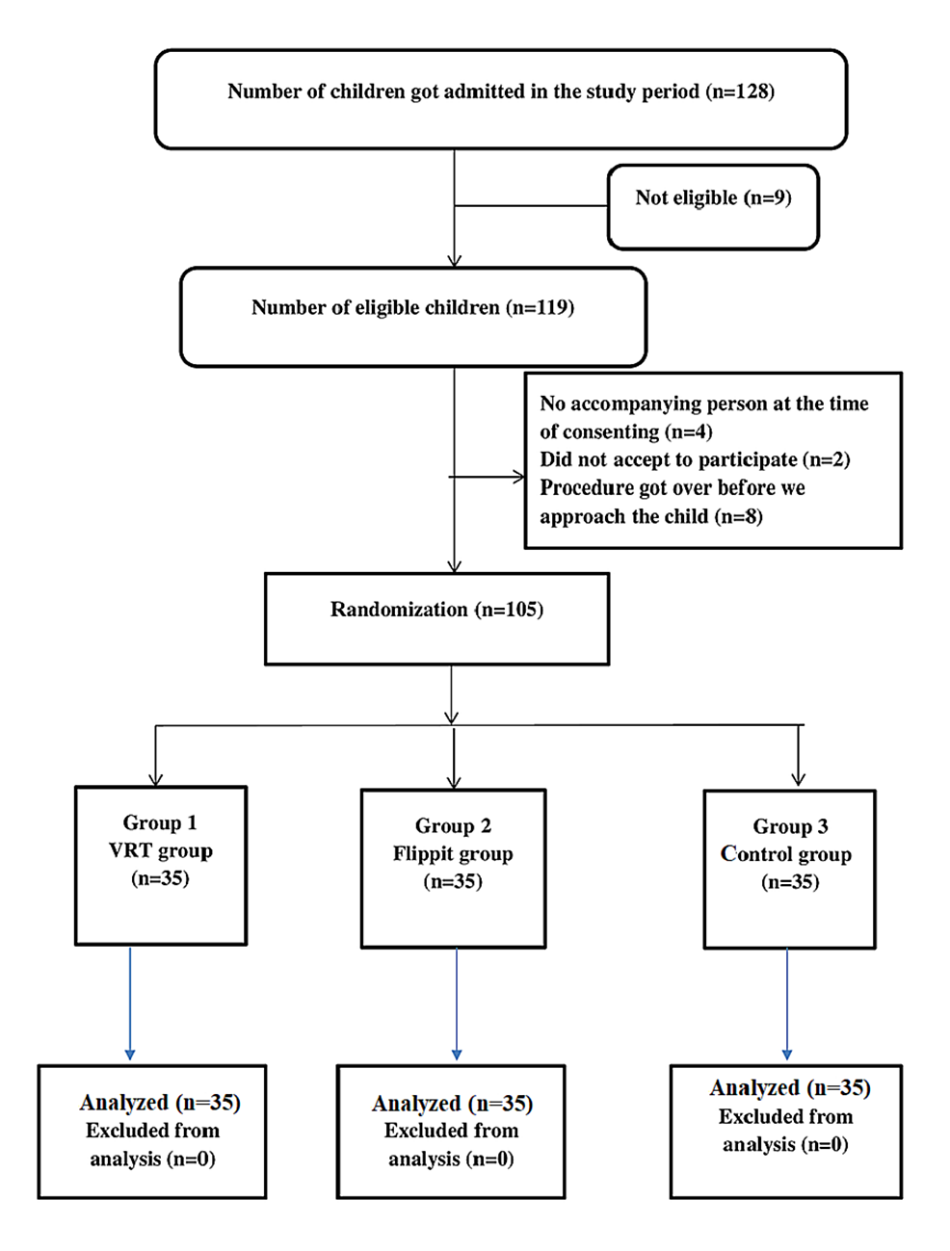
CONSORT diagram showing the flow of participants CONSORT: Consolidated Standards of Reporting Trials; VRT: virtual reality therapy

We included 105 children, aged between three years and 12 years, who were admitted to the pediatric ward, undergoing painful procedures such as IV cannulation, blood sampling, and intramuscular injections for the first time after admission (Figure 1). Children, who were sick, mentally retarded, had hearing and visual impairment and received painful procedures under local or general anesthesia were excluded.

Randomization and Blinding

Included children were randomized to either one of three groups (flippit, VRT, or control group). The randomization sequence was generated by a web-based random number sequence generator (www.randomization.com). Separate person, who did not involve in the study, generated the random sequence. Participants were allocated using variable block sizes (three and five) to three groups. Allocations were concealed by placing the allocation sequence in opaque, tamper-proof, sealed, serially numbered envelopes. Participants and the primary investigator (intervention therapist) were not blinded due to the nature of the intervention. But the statistician was blinded from the study allocation details. Attempts were made to minimize the bias by maintaining a strict study protocol.

Interventions

Children allocated to group 1 received VRT. We used GKP PRODUCTS VR-Box, Model 223741 (GKP products, India), which contains virtual reality 3D glasses for smartphones, to deliver the VRT. The smartphone (iPhone XR {Foxconn, Taiwan}) was prerecorded with five famous cartoon comic videos which are favorite to children. Those videos were chosen by conducting a survey among children less than 12 years of age. Children were allowed to get familiar with the virtual reality box for 15 minutes before the painful procedure, and they were asked to choose one of their interested videos out of the five prerecorded videos to be played for them during the procedure. The video was displayed through a head-mounted display and a thick pair of goggles connected with the smartphone during the time of painful procedure until the completion of the procedure. Once the procedure was over, the child was instructed to remove the display and get into the real world. The VRT equipment was thoroughly cleaned using 70% isopropyl alcohol disinfectant after the use by each patient and cleaned with a dry cloth just before use to the next one. In order to avoid discomfort to children due to the smell of isopropyl alcohol, we made sure to wait till the smell of isopropyl alcohol disappears before using on a child. 

Children allocated to group 2 received flippit therapy, in which visual cards of 9 inch width and 12 inch length contain famous cartoon characters were shown during the painful procedure. The cartoon characters which were used in flippit therapy were similar to VRT. The researcher interacted with children and encouraged them to tell stories behind that particular cartoon character while flipping the cards during the painful procedure. To preserve the feel of thrill, it was planned to show the video and flippit cards only after entering into the procedure room and 5 minutes before the painful procedure, not during the time of instruction and before entering into the procedure room. The painful procedure was started after 5 minutes of intervention and the interventions were continued throughout the procedure. Children allocated to group 3 (control group) received conventional measures such as psychological support, verbal diversion, and storytelling during the painful procedure.

Outcomes

**Figure 2:**
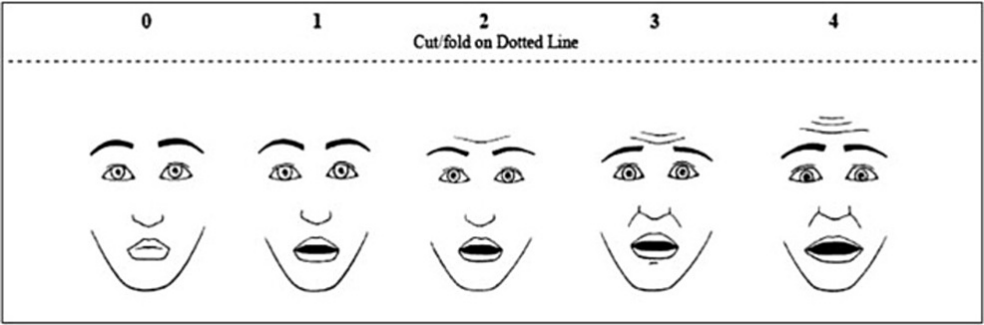
Children’s Fear Scale Source: Adapted from the Faces Anxiety Scale [12].

Demographic variables were collected using the structured data collection form. All the children were assessed for anxiety after 5 minutes of starting VRT or flippit in intervention groups, just before the painful procedure in the control group. Pain, heart rate, respiratory rate, and saturation were assessed during the procedure in all three groups. Children’s pain was measured by researchers using the standard Wong-Baker Faces Scale which has score of 0 to 10 and increasing intensity of pain with the increasing scores [10]. Score 0 meant for no hurt, score of 2, 4, 6, 8, 10 meant for hurts a little bit, little more, even more, whole lots, and worst, respectively. Anxiety was also measured by researchers using the Children’s Fear Scale which has score of 0 to 4 and increasing intensity of anxiety with the increasing score (Figure 2) [11]. Both pain and anxiety were measured by two researchers individually. The measurements were confirmed with children and accompanying persons. The final decision was left with children. Heart rate and saturation were measured by using Masimo Radical 7 Pulse Oximeter (Masimo Corporation, USA). Respiratory rate was measured by counting the respiration for one minute. The respiratory rate was counted after the children stop their cry. 

Sample size

The standard deviation of pain in the VRT group and flippit group from our unpublished previous studies were 1.34 and 1.7, respectively. The mean difference was 0.72. Desired confidence level and power were taken as 95% and 80%, respectively. The calculated sample using N master software (Department of Biostatistics, Christian Medical College, Vellore) was 35 per group. The total sample size of our study was 105 for three groups. A simple random sampling technique was used to recruit the study population. 

Statistical analysis

Descriptive statistics were used to describe baseline variables. Categorical outcome variables were analyzed by chi-square test with continuity correction or Fisher’s exact test, wherever one or more expected cell sizes was less than five. Numerical variables were first tested for normality utilizing the Kolmogorov-Smirnov test. Continuous variables were analyzed using the Kruskal-Wallis test as data were not normally distributed. Multiple logistic regression analysis was done to adjust for potential confounding variables. The choice of variables to be adjusted was based on biological plausibility and/or on the statistical significance (P < 0.20) of their association with the outcome of interest in the univariate analysis. The correlation coefficient was assessed using Spearman's Rho correlation formula. An intention-to-treat (ITT) analysis was done. All analysis was done using SPSS version 23 (Armonk, NY: IBM Corp.). P-value of less than 0.05 was considered significant.

## Results

Total 128 children were admitted to the ward and 23 were not included in the study for various reasons as mentioned in Figure 1. Total 105 children undergone randomization to three groups, 35 in each group. All were analyzed for primary and secondary outcomes. The majority of the children were accompanied by mothers in all the groups (65.7%, 51.4%, and 45.7% in VRT, flippit, and control groups, respectively). Majority of children were aged between nine to 12 years in VRT and control groups (48.6%) and six to nine years in flippit group (45.7%). Children aged three to six years were more in the flippit group (42.9%) than VRT (25.7%) and control group (22.8%). Male children were more in flippit group and control group (60% and 71.4 %, respectively) than the VRT group (37.1%). Most of the children had an experience of pain (82.9%, 74.3%, and 85.7% in VRT, flippit, and control groups, respectively) due to various painful procedures such as immunization, intravenous cannulation, and blood sampling, etc. within one year of this admission. The age and gender of children were not equally distributed among the three groups (P = 0.007 and P = 0.046, respectively; Table 1). 

**Table 1:** Baseline characters *P-value is significant at 0.05. **Pain due to injection, intravenous cannulation, and blood sampling. VRT: virtual reality therapy

Variables	Frequency (n, %)			Chi-square (P-value)
	VRT group (N=35)	Flippit group (N=35)	Control group (N=35)	
Accompanying person				0.099
Father	7 (20)	16 (45.7)	15 (42.9)	
Mother	23 (65.7)	18 (51.4)	16 (45.7)	
Other	5 (14.3)	1 (2.9)	4 (11.4)	
Age of the child				0.007*
3-6 years	9 (25.7)	15 (42.9)	8 (22.8)	
6-9 years	9 (25.7)	16 (45.7)	10 (28.6)	
9-12 years	17 (48.6)	4 (11.4)	17 (48.6)	
Gender of the child				0.046*
Male	13 (37.1)	21 (60)	25 (71.4)	
Female	22 (62.9)	14 (40)	10 (28.6)	
Experience of pain within one year of this admission**				0.448
Yes	29 (82.9)	26 (74.3)	30 (85.7)	
No	6 (17.1)	9 (25.7)	5 (14.3)	

There was a difference in perception of pain and anxiety among the three groups (P = 0.000 and P = 0.006, respectively; Table 2).

**Table 2:** Comparison of perception of pain and anxiety among the three groups *P-value is significant at 0.05. VRT: virtual reality therapy

Primary outcome	Overall score (median, IQR)			Kruskal-Wallis test
	VRT group (N=35)	Flippit group (N=35)	Control group (N=35)	P-value
Pain	3 (3-4)	1 (1-4)	5 (3-10)	0.000*
Anxiety	2 (1-3)	1 (1-2)	2 (1-3)	0.006*

There was a difference in intensity of pain among the three groups (P = 0.000; Table 3).

**Table 3:** Comparison of the intensity of pain among the three groups *P-value is significant at 0.05. VRT: virtual reality therapy

Intensity of pain	VRT group (N=35)	Flippit group (N=35)	Control group (N=35)	Chi-square (P-value)
No hurt	0	0	0	0.000*
Hurts little	20	26	13	
Hurts more	14	1	9	
Hurts lot	1	1	4	
Hurts worst	0	7	9	

Univariate regression analysis was done, confounding variables were identified for primary outcomes and they were adjusted by multivariate regression analysis. After adjusting for confounding factors using multiple logistic regression, it was found that pain scores of VRT and flippit groups were less than the control group (aOR, 95% CI 0.635, 0.504-0.799, P = 0.000 and aOR, 95% CI 0.705, 0.572-0.868, P = 0.001, respectively) and no difference was observed between VRT and flippit groups (aOR, 95% CI 0.901, 0.723-1.123, P = 0.353). Flippit group perceived less intensity of pain compared to control group (aOR, 95% CI 0.542, 0.322-0.912, P = 0.021) and children received VRT perceived less intensity of pain than both control and flippit groups of children (aOR, 95% CI 0.258, 0.132-0.503, P = 0.000 and aOR, 95% CI 0.476, 0.252-0.900, respectively). Children received VRT and flippit therapy perceived less anxiety compared to control group (aOR, 95% CI 0.589, 0.348-0.999, P = 0.050 and aOR, 95% CI 0.385, 0.217-0.682, P = 0.001, respectively). But there was no difference between VRT and flippit groups (aOR, 95% CI 1.532, 0.940-2.498, P = 0.087; Table 4). 

**Table 4:** Comparison of primary outcomes among the three groups after adjusting for confounding factors using multiple logistic regression analysis *Adjusted for age and previous experience. **Adjusted for age. ***Adjusted for age and gender. ****P-value is significant at 0.05. VRT: virtual reality therapy

Comparison groups	Overall pain score*			Intensity of pain^**^			Overall anxiety score^***^		
	aOR	95% CI	P-Value	aOR	95% CI	P-Value	aOR	95% CI	P-Value
VRT vs control	0.635	0.504-0.799	0.000****	0.258	0.132-0.503	0.000****	0.589	0.348-0.999	0.050
Flippit vs control	0.705	0.572-0.868	0.001****	0.542	0.322-0.912	0.021****	0.385	0.217-0.682	0.001****
VRT vs flippit	0.901	0.723-1.123	0.353	0.476	0.252-0.900	0.022****	1.532	0.940-2.498	0.087

**Figure 3:**
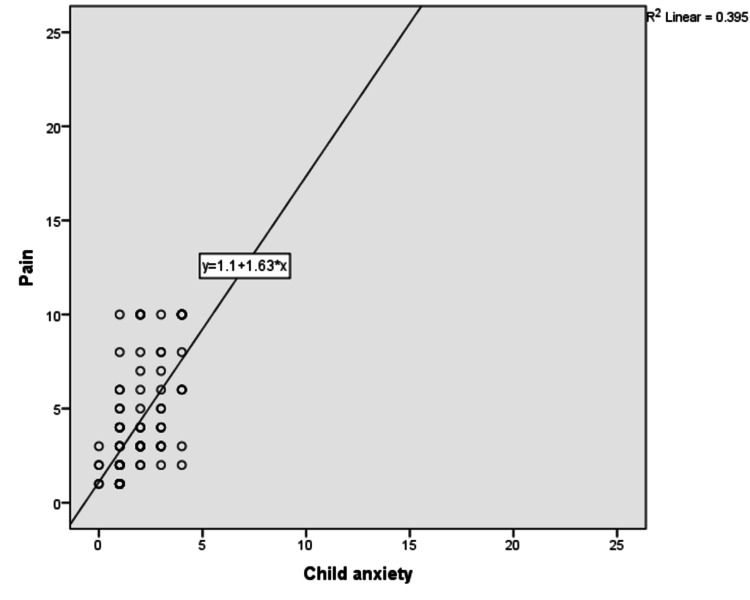
Correlation between child anxiety and pain

**Figure 4:**
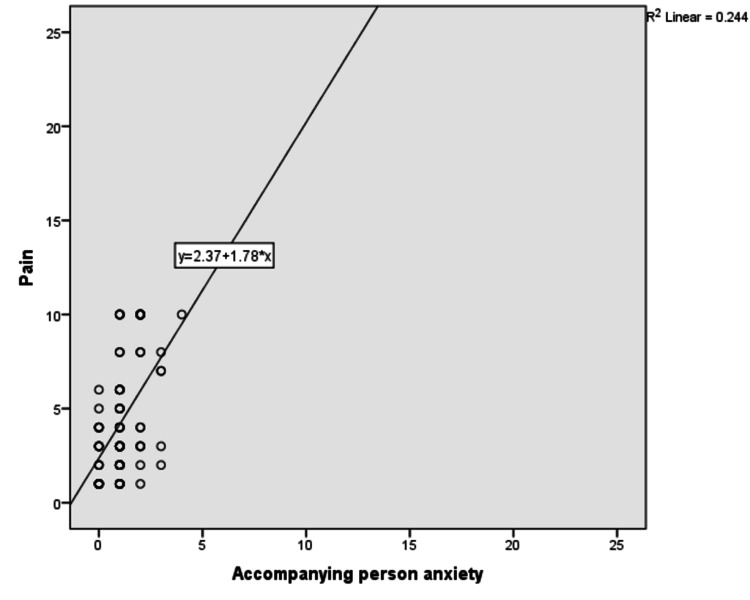
Correlation between child’s pain and accompanying person anxiety

**Figure 5:**
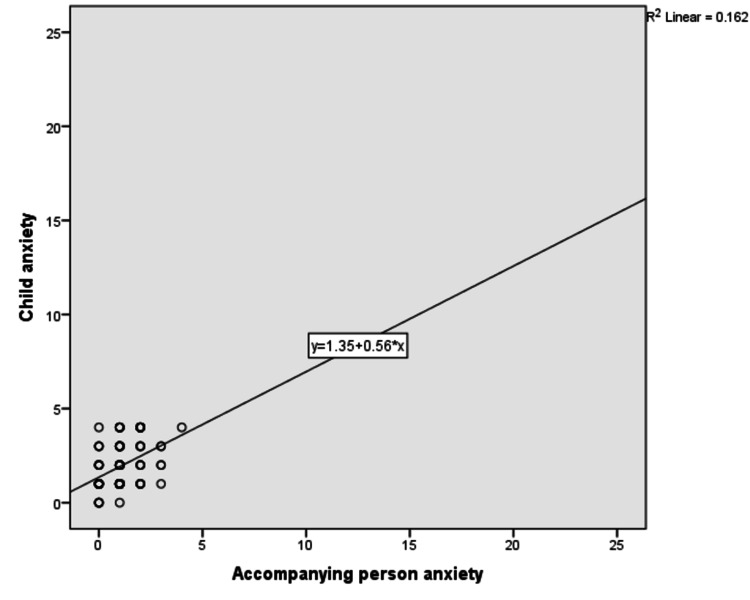
Correlation between child’s anxiety and accompanying person anxiety

We found that there was a low positive correlation between children’s anxiety and their pain (r^2^ = 0.395; Figure 2), but there was a negligible correlation between accompanying person’s anxiety and children’s anxiety and pain (r^2^ = 0.244 and 0.162, respectively; Figures 3-5). 

There was a significant difference in heart rate and respiratory rate median score among the three groups (P = 0.001 and P = 0.000, respectively; Table 5).

**Table 5:** Effect of interventions on secondary outcomes *P-value is significant at 0.05. VRT: virtual reality therapy

Secondary outcomes	Overall score (median, IQR)			Kruskal-Wallis test
	VRT group (N=35)	Flippit group (N=35)	Control group (N=35)	P-value
Heart rate	82.00 (78-86)	80.00 (76 – 84)	85.00 (82 – 92)	0.001*
Respiratory rate	22.00 (22-24)	20.00 (20 – 22)	24.00 (22 – 26)	0.000*

## Discussion

This randomized control trial (RCT) studied the effect of VRT and flippit therapy on pain and anxiety of children aged three to 12 years undergoing painful procedures compared to the control group. This trial found that VRT and flippit therapy were better than the control group in reducing the perception of pain, intensity of pain, and perception of anxiety. We also found that compared to flippit therapy, VRT was better one for reducing the intensity of pain. Heart rate and respiratory rate which were measured during the painful procedure were more in the control group compared to VRT and flippit therapy groups which might be due to more pain and anxiety scores in the control group than intervention groups.

RCTs done by Risaw et al. and Canbulat et al. showed that distraction technique using flippit cards can significantly reduce pain associated with blood sampling in children compared to the control group and compared to control/Kaleidoscope group, respectively [13,14]. With the best of our knowledge, we could find only one published RCT compared distraction card (flippit card) versus VRT. Ours' is the next one that compared VRT and flippit therapy and found that VRT was better than flippit therapy as no children perceived the worst pain in the VRT group, but in flippit group. The RCT done by Erdogan et al. among the children aged seven to 12 years showed that the mean (SD) value of pain in flippit and VRT groups were 1.6 ± 1.3 and 0.8 ± 0.9 (P = 0.05), respectively [15]. This supports our finding of the superiority of VRT on reducing the pain in children undergoing painful procedures over flippit therapy. A meta-analysis, done by Eijlers et al., of 14 studies for pain and anxiety which included participants from four to 21 years of age proved the efficacy of VRT than care as usual (CAU). This meta-analysis showed that VRT was potentially more efficacious for younger than for older children [16].

High-intensity anxiety of children is associated with high intensity of pain [17]. Our study also showed a positive correlation between children’s anxiety and pain. We found that children who received VRT and flippit therapy had less anxiety than the control group, but did not find any superiority between them. RCT done by Erdogan et al. also found the advantage of VRT and flippit therapy (distraction card) over the control group in reducing children’s anxiety, but did not find difference between VRT and flippit therapy [15]. In our study, we found that the accompanying person’s anxiety had a negligible correlation with children’s anxiety and pain. However, Esteve et al. proved that caregivers’ anxiety was positively correlated with the child’s anxiety [17]. Our study showed the effectiveness of VRT over flippit therapy on common procedural pain such as pain due to intravenous cannulation, blood sampling, and intramuscular injections. VRT was checked widely as a distraction technique to reduce the perception of pain for various procedures such as intravenous cannulation, dental, burn, and oncological care and for lumbar puncture, etc. [13,14]. Unless other analgesics act by interrupting the C-fiber pathway, VRT acts on pain perception, attention, emotion, concentration, memory, and other senses [18]. It was also proved that VRT significantly reduced pain-related brain activity in the anterior cingulate cortex, primary and secondary somatosensory cortex, insula, and thalamus [19].

We used famous cartoon comics as a part of an intervention to entertain the children in both VRT and flippit therapy. The content chosen were similar in both the groups (VRT and flippit) in order to avoid bias due to the variation in the content of entertainment. Many more virtual reality applications were used by other researchers such as SnowWorld (exploring an icy three-dimensional world throwing snowballs at snowmen, penguins, and woolly mammoths), Gorilla Exhibit (visiting the gorilla habitat at Zoo Atlanta), Aqua (exploring and interacting with a comfortable underwater world), Bear Blast (shooting balls at animated teddy bears), Feeding Frenzy (launching different types of food to hungry animals), and Shape Your Path (matching colored bricks while avoiding obstacles to reach a destination) [20].

In our study, we used the age-appropriate tool for assessment of pain (Wong-Baker FACES® Pain Rating Scale) and anxiety (Children’s Fear Scale). Even though we could not blind the patients and outcome assessors due to the nature of interventions, we took the effort to reduce the bias in the assessment of primary outcomes by involving two researchers, accompanying persons, and children to measure. As both pain and anxiety were assessed using a facial expression scale, it was decided to measure one outcome variable at a single point of time, unlike other studies. As the pain will be maximum during the procedure and the pre-procedural anxiety will influence the pain (direct proportion) during the procedure, we decided to measure the anxiety before the painful procedure and the pain during the procedure. We believed that any intervention which is effective in reducing the pain during the procedure will be effective in reducing the post-procedural pain also and this was supported by other researchers [10-11,13].

The cost spent for VRT was slightly higher compared to flippit therapy (INR 500 vs 100, respectively) but affordable. The need to take care of infection control measures for VRT as the equipment used is common for all children, but it will not be a major problem in flippit therapy. Children under VRT may react to the video and make frequent body movements during the medical procedure compared to flippit therapy. Children need more time to get familiarize with VRT equipment than flippit therapy and hence the therapist needs to spend more time with children well before the procedure starts. 

Strengths and limitations

Major strengths of this study are its robust study design, used standard tools for assessment of outcomes, efforts were taken to reduce the bias due to interobserver variability in outcome assessment, content used to entertain the children was same in both the groups to avoid the bias due to content variability, efficacy of interventions were tested for most common procedures, studied in a wide range of age group (three to 12 years), and efficacy of VRT was proved with low-cost virtual reality device. Major limitation of this study was the small sample size.

## Conclusions

Children’s pre-procedural anxiety had a positive correlation with their pain. Flippit therapy and virtual reality therapy were better than conventional measures in reducing the perception of anxiety and pain in children, aged three to 12 years, undergone painful procedures. Virtual reality therapy had an edge over flippit therapy in reducing the worst hurt. These interventions can be used in children undergone intravenous cannulation, blood sampling, and injections like immunization. This study should be replicated with a large sample size to confirm these results. Distracting attention from pain with the help of virtual reality or distraction card (flippit), particularly in children, may help nurses to work more efficiently. It could be better to do similar kind of studies in children aged below three years as they are more vulnerable population for pain and anxiety.
